# Metagenomic Investigation of Intestinal Microbiota of Insectivorous Synanthropic Bats: Densoviruses, Antibiotic Resistance Genes, and Functional Profiling of Gut Microbial Communities

**DOI:** 10.3390/ijms26135941

**Published:** 2025-06-20

**Authors:** Ilia V. Popov, Andrey D. Manakhov, Vladislav E. Gorobets, Kristina B. Diakova, Ekaterina A. Lukbanova, Aleksey V. Malinovkin, Koen Venema, Alexey M. Ermakov, Igor V. Popov

**Affiliations:** 1 Faculty “Bioengineering and Veterinary Medicine”, Don State Technical University, 344000 Rostov-on-Don, Russia; 2 Division of Genetics, Center for Genetics and Life Science, Sirius University of Science and Technology, 354340 Sirius, Russia; 3 Vavilov Institute of General Genetics, Russian Academy of Sciences, 119991 Moscow, Russia; 4 Beneficial Microbes^®^ Consultancy, 6709 TN Wageningen, The Netherlands; 5 Division of Immunobiology and Biomedicine, Center of Genetics and Life Sciences, Sirius University of Science and Technology, 354340 Sirius, Russia

**Keywords:** bats, gut microbiota, metagenomics, phylogenomics, microbial ecology, One Health

## Abstract

Bats serve as key ecological reservoirs of diverse microbial communities, including emerging viruses and antibiotic resistance genes. This study investigates the intestinal microbiota of two insectivorous bat species, *Nyctalus noctula* and *Vespertilio murinus*, at the Rostov Bat Rehabilitation Center in Southern Russia using whole metagenome shotgun sequencing. We analyzed taxonomic composition, functional pathways, antibiotic resistance genes, and virulence factors. Densoviruses, especially those closely related to *Parus major densovirus*, were the most dominant viral sequences identified. Metagenome-assembled densovirus genomes showed high sequence similarity with structural variations and clustered phylogenomically with viruses from mealworms and birds, reflecting both dietary origins and the potential for vertebrate infection. Functional profiling revealed microbial pathways associated with cell wall biosynthesis, energy metabolism, and biofilm formation. A total of 510 antibiotic resistance genes, representing 142 unique types, mainly efflux pumps and β-lactamases, were identified. Additionally, 870 virulence factor genes were detected, with a conserved set of iron acquisition systems and stress response regulators across all samples. These findings highlight the ecological complexity of bat-associated microbiota and viromes and suggest that synanthropic bats may contribute to the circulation of insect-associated viruses and antimicrobial resistance in urban settings.

## 1. Introduction

Chiroptera, the second most diverse order of mammals after rodents, includes more than 1400 species and represents approximately 20% of the world’s mammal population [1]. These animals play a crucial role in the global microbial ecology and One Health, serving as natural reservoirs for an extensive diversity of microorganisms [2,3,4]. Unique physiological features contribute to their exceptional ecological significance, including their ability to fly, efficient immune system, and metabolic adaptations that allow them to asymptomatically coexist with numerous pathogens [5,6]. Their ability to fly, combined with a lifespan significantly longer than that of other mammals of similar size, enables bats to facilitate the widespread dissemination of microorganisms across diverse environments over both spatial and temporal scales [7]. These features also contribute to the spread of antibiotic resistance genes, posing a significant challenge to both public health and animal welfare [8]. As bats interact with diverse ecosystems, their movements can unexpectedly facilitate the transfer of these genes between microbial communities, potentially impacting the production performance of farm animals and the effectiveness of antibiotic therapy [9]. Consequently, bats can be viewed as “living bioreactors” for microbial and antibiotic resistance gene spillovers [7].

The most infamous examples of the role of bats in microbial dynamics include their involvement in emerging pathogens, such as coronaviruses (e.g., SARS-CoV, MERS-CoV, and SARS-CoV-2), Ebola virus, Nipah virus, and Marburg virus [10]. Although there are no definitive conclusions on whether bats were the original hosts for some of the progenitors of these viruses, the detection of homologous viruses closely related to those responsible for epidemics and pandemics in bat populations underscores their involvement in zoonotic transmission chains [11,12].

The ecological role of bats extends far beyond their interactions with various microbial communities. Based on their dietary preferences, bats serve as pollinators (nectarivorous species), seed dispersers (frugivorous species), and regulators of insect populations (insectivorous species) [13,14,15]. These functions are vital for maintaining ecological balance and supporting the balance of agricultural and urban environments, ultimately contributing to the economy and One Health [16,17]. The influence of bats on the economy, agriculture, and healthcare is evident in the connection between bat population dynamics, agricultural productivity, and infant mortality rates. While the relationship between bats and agriculture is obvious, the impact of bat populations on infant mortality rates has only recently been established. Eyal G. Frank demonstrated that the loss of bat biodiversity caused by white-nose syndrome, one of the deadliest infections in bats, leads to a significant increase in the use of insecticides to compensate for the decline in natural pest control provided by bats, which in turn affects the quality of food and subsequently human infant health [18]. The causative agent of the white-nose syndrome is *Pseudogymnoascus destructans*, which infects the skin of hibernating bats, which are insectivorous and contribute to pest control [19]. Fungi belonging to the *Pseudogymnoascus* genus are usually isolated from soil or plants, which suggests that the progenitor of *P. destructans* originated from the environment [20]. Also, *P. destructans* can persist and grow in environmental substrates like soil, wood, and guano, which supports the hypothesis that it originated from environment-associated fungi [21]. Thus, these findings demonstrate that bats and their associated microbial communities are deeply integrated into both ecological and One Health frameworks, underscoring the need for comprehensive research and thoughtful interpretation of the results.

In this study, we aimed to assess the intestinal microbial communities of common noctules (*Nyctalus noctula*) and parti-colored bats (*Vespertilio murinus*) from the Rostov Bat Rehabilitation Center, Rostov-on-Don, Russia. These bats are insectivorous and abundant in the Rostov region, especially *N. noctula*, which, according to the bat rehabilitation data, is the most dominant species in urban areas. The gut microbiota composition of these bat species was previously studied based on V3-V4 16S rRNA metaprofiling data, which has limitations in terms of selective analysis of composition and diversity of bacteria (and archaea), while completely excluding the investigation of other representatives of microbial communities, including viruses and fungi, and their genomic features [22]. This study is the continuation of microbial ecology studies of synanthropic bats, and is focused on a deeper investigation of metagenomic signatures of their intestinal microbes.

## 2. Results

### 2.1. Microbial Community Composition and Diversity Analysis

**Figure 1:**
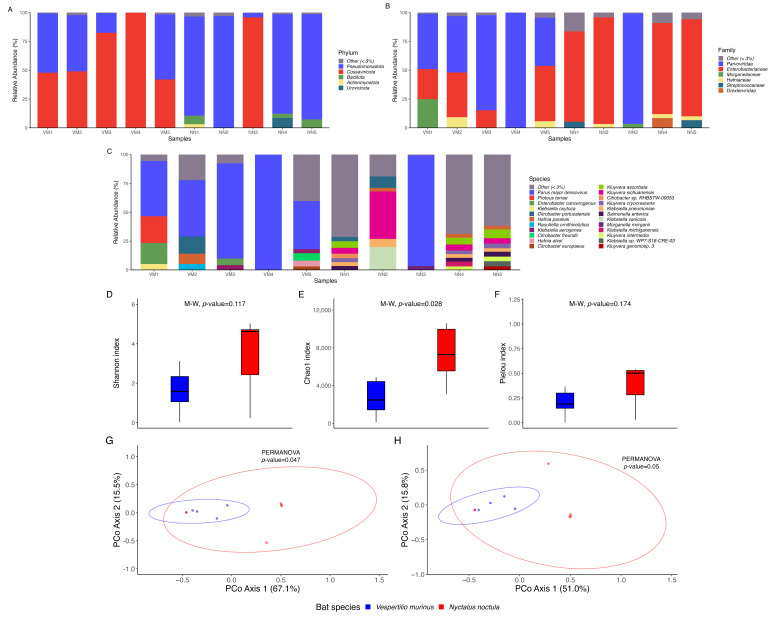
Taxonomic composition and diversity metrics of bat gut microbiota. Relative abundance of microbial taxa at the phylum level (**A**), family level (**B**), and species level (**C**) across 10 metagenomic samples. Samples VM1–VM5 correspond to *Vespertilio murinus*, while NN1–NN5 represent *Nyctalus noctula*. (**D**)—Shannon index, (**E**)—Chao1 richness index, (**F**)—Pielou’s evenness index (alpha diversity). (**G**)—Bray–Curtis dissimilarity, (**H**)—Jaccard index (beta diversity).

Taxonomic profiling revealed the relative abundance of microorganisms in each sample (Figure 1A–C). Notably, *Parus major densovirus* dominated the microbial composition in two samples (NN3 and VM4), accounting for over 90% of the total sequences (Figure 1C), and was also highly abundant (>40%) in four additional samples (VM1–VM3, VM5). On the phylum level (Figure 1A), despite the marked dominance of the *Cossaviricota*, to which *Parus major densovirus* belongs, the microbial communities were largely composed of *Pseudomonadota* across most samples. *Bacillota*, *Actinomycetota*, and *Uroviricota* were also present in some samples in lower relative abundances. Family-level analysis (Figure 1B) of the gut microbial communities revealed a notable dominance of *Parvoviridae* and *Enterobacteriaceae* alongside lower abundances of *Morganellaceae*, *Hafniaceae*, *Streptococcaceae*, and *Drexlerviridae*. On the species level (Figure 1C), the intestinal microbial communities of the studied bat species were composed of multiple taxa, such as *Proteus terrae*, *Enterobacter cancerogenus*, *Klebsiella oxytoca*, *Citrobacter portucalensis*, *Hafnia paralvei*, *Raoultella ornithinolytica*, *Klebsiella aerogenes*, *Citrobacter freundii*, *Hafnia alvei*, *Citrobacter europaeus*, *Kluyvera ascorbata*, *Kluyvera sichuanensis*, *Citrobacter* sp. RHBSTW-00053, *Kluyvera cryocrescens*, *Klebsiella pneumoniae*, *Salmonella enterica*, *Klebsiella variicola*, *Morganella morganii*, *Klebsiella michiganensis*, *Kluyvera intermedia*, *Klebsiella* sp. WP7-S18-CRE-03, *Kluyvera genomosp*. 3, and others. However, as mentioned earlier, the overall species composition in the studied samples was overwhelmingly dominated by *Parus major densovirus*, which skewed the abundance of other representatives of the studied microbial communities.

Alpha diversity analysis did not reveal relevant differences between the two bat species (*V. murinus* and *N. noctula*). The Shannon (*p* = 0.117) (Figure 1D) and Pielou (*p* = 0.174) (Figure 1E) indices indicated no substantial variation in overall diversity and evenness, while Chao1 (*p* = 0.028) (Figure 1D) suggested a difference in species richness. Beta diversity analysis showed a weak but statistically significant effect. PERMANOVA results for Bray–Curtis (*p* = 0.047) (Figure 1E) and Jaccard (*p* = 0.05) (Figure 1F) distances suggested some level of community structure differentiation.

Differential abundance analysis did not detect statistically significant taxonomic shifts between the two groups at any of the tested taxonomic levels (Figures S1–S6).

### 2.2. Functional and Metabolic Profiling

**Figure 2:**
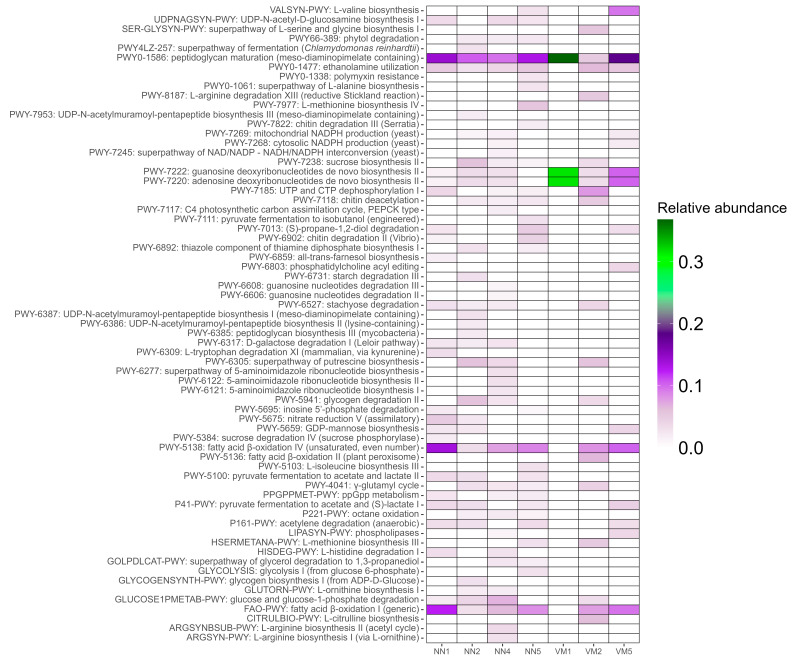
Functional potential of bat gut microbiota based on metabolic pathway abundance. Heatmap of relative abundances of metabolic pathways predicted by HUMAnN3. Rows are individual pathways predicted by HUMAnN3; columns are samples (NN1–NN5 = *Nyctalus noctula*, VM1–VM5 = *Vespertilio murinus*). Color intensity represents normalized relative abundance (each column sums to 1).

Annotation of metabolic pathways using HUMAnN3 identified 67 pathways across seven samples, while no pathways were annotated in three samples (NN3, VM3, and VM4). The heatmap (Figure 2) of relative pathway abundance highlighted peptidoglycan maturation as the most prevalent across all annotated samples, suggesting a significant presence of actively growing bacteria with cell walls and their role in microbial interactions. The VM1 sample exhibited the lowest functional diversity, with only three pathways detected: peptidoglycan maturation, guanosine deoxyribonucleotides de novo biosynthesis II, and adenosine deoxyribonucleotides de novo biosynthesis II. This result aligns with taxonomic profiling and biodiversity assessment, which revealed low species diversity in the VM1 sample, consisting of only four taxa: *Parus major densovirus*, *Proteus terrae*, *Enterobacter cancerogenus*, and *Klebsiella oxytoca*. Differential pathway abundance analysis did not yield statistically significant results (Figure S7).

**Figure 3:**
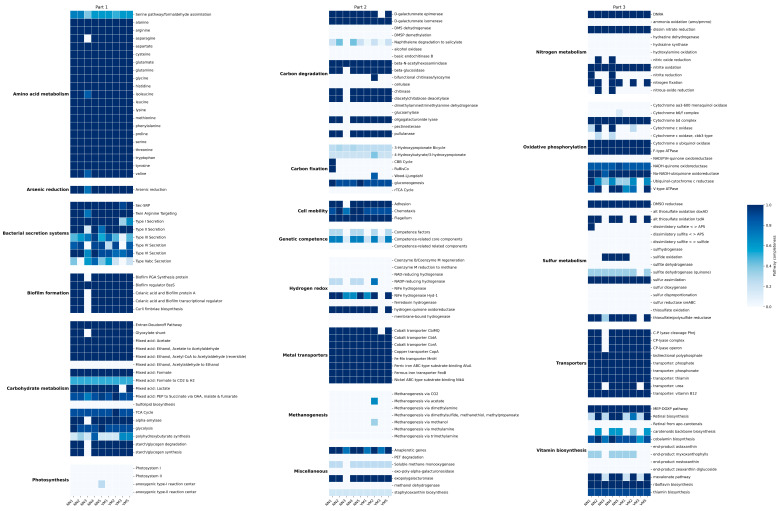
Completeness of KEGG metabolic pathways in bat gut microbiota. Heatmap displaying the completeness of over 170 individual KEGG pathways, divided into three nearly equal sections for clarity. Each cell corresponds to a specific metabolic function, labeled on the right. Functions are grouped by category, with group names indicated on the left. Gaps separate different functional groups. Columns represent samples (NN1–NN5 = *Nyctalus noctula*, VM1–VM5 = *Vespertilio murinus*).

**Figure 4:**
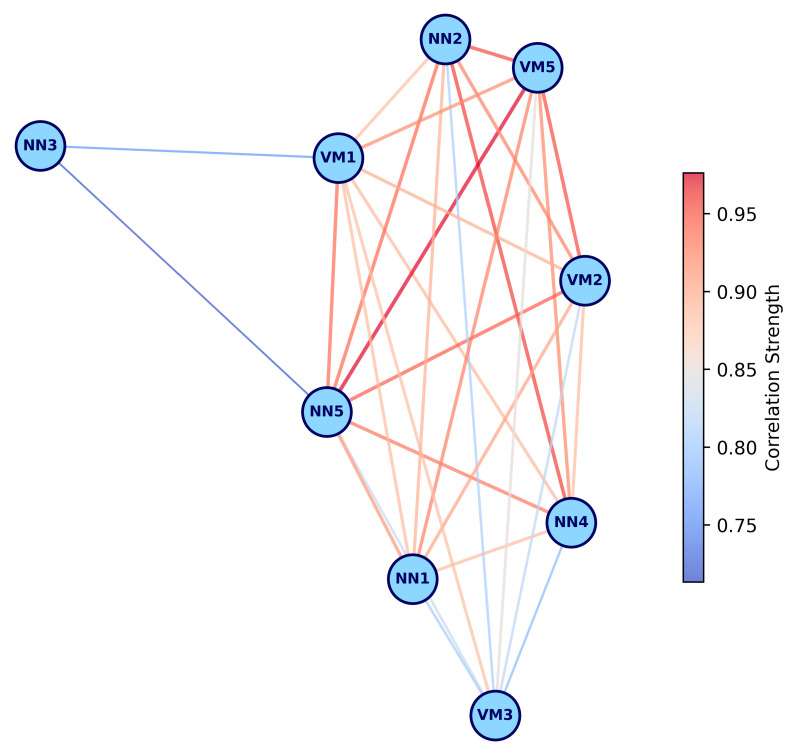
KEGG metabolic profile correlation network. Network illustrating correlations greater than 0.7, with weak correlations shown in blue and strong correlations in red. The thickness of the lines increases with the strength of the correlation.

Further annotation of KEGG metabolic pathways using eggNOG-mapper revealed six major functional categories consistently represented across most samples (Figure 3): (i) amino acid metabolism, (ii) carbon degradation, (iii) nitrogen and sulfur metabolism, (iv) oxidative phosphorylation, (v) bacterial secretion systems, and (vi) biofilm formation. These results indicate active microbial participation in amino acid turnover, efficient organic matter processing, and involvement in biogeochemical nitrogen and sulfur cycles, alongside robust energy production via aerobic respiration. The presence of bacterial secretion systems and biofilm formation pathways aligns with the HUMAnN3 annotation, reinforcing the validity of both analyses. The NN3 sample exhibited the lowest functional annotation completeness in the KEGG pathway analysis, while VM4 had no annotated pathways. The distinctiveness of NN3 was further supported by correlation network analysis of sample relationships (Figure 4).

### 2.3. Antimicrobial Resistance and Virulence Genes

**Figure 5:**
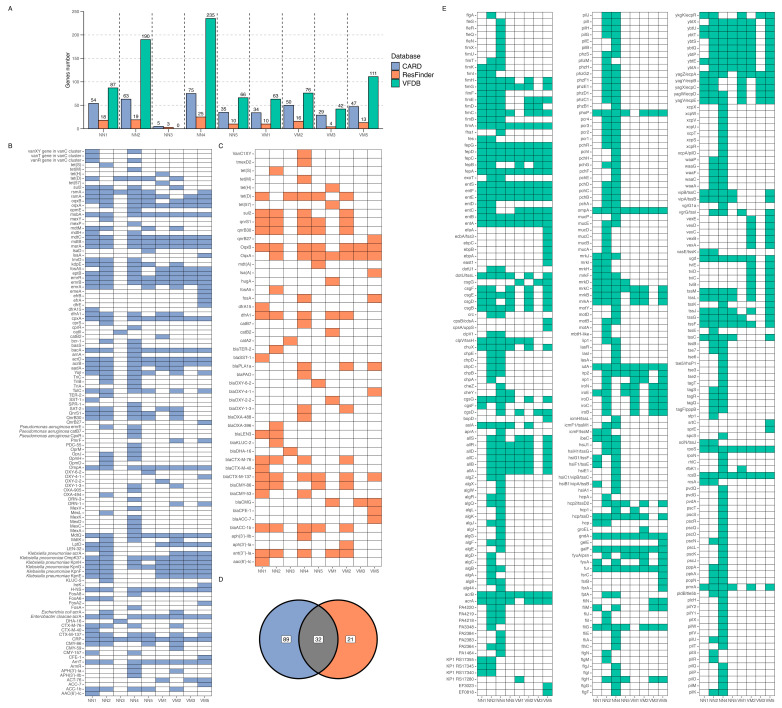
Identification and presence of antibiotic resistance genes and virulence factors across metagenomic samples. (**A**)—Bar plot of gene counts from CARD, ResFinder, and VFDB databases identified in each sample. Blue bars represent CARD genes, orange bars represent ResFinder genes, and green bars represent VFDB genes. A black dashed line separates the samples. (**B**,**C**)—Presence tables for CARD and ResFinder genes, respectively, across the metagenomic samples. (**D**)—Vienn diagram showing overlapping and non-overlapping identifications of antibiotic resistance genes by CARD (blue) and ResFinder (orange) databases. (**E**)—Presence tables for VFDB genes across the metagenomic samples. X-axis labels on bar plot (**A**) and columns in heatmaps (**B**,**C**,**E**) represent samples (NN1–NN5 = *Nyctalus noctula*, VM1–VM5 = *Vespertilio murinus*).

Screening for ARGs across ten samples resulted in a total of 510 detections using the CARD and ResFinder databases (Figure 5A–C). In total, 142 unique ARGs were identified across the dataset: 121 from CARD and 53 from ResFinder, with 32 genes detected by both databases (Figure 5D). Virulence factor identification using the VFDB database revealed a total of 870 gene detections across the ten samples (Figure 5A). These correspond to 314 unique virulence factors identified across the entire dataset (Figure 5E). The NN3 sample exhibited the lowest ARG count, with only eight detected (five from CARD and three from ResFinder), whereas VM4 lacked any annotated resistance genes.

Among the ARGs (Figure 5B), *CRP* was the only one present in all nine annotated samples. This global regulator represses the MdtEF multidrug efflux pump and is associated with resistance to penicillins, fluoroquinolones, and macrolides. Additionally, 17 genes were consistently detected in eight samples, primarily encoding efflux pumps from the resistance-nodulation-cell division (RND) family (*mdtC*, *mdtB*, *acrD*, *acrB*), ATP-binding cassette transporters (*msbA*), and porins with reduced permeability (*marA*, *OmpA*, *MdtQ*). These genes mediate resistance to a broad spectrum of antibiotics, including aminoglycosides, beta-lactams, tetracyclines, fluoroquinolones, and carbapenems. The major facilitator superfamily efflux system was also well represented, with *emrR*, *emrB*, and *Klebsiella pneumoniae KpnH*/*KpnG* contributing to resistance against macrolides, cephalosporins, and peptide antibiotics.

A smaller subset of ARGs was found in seven out of nine samples, including *ramA* (a regulator affecting multiple resistance mechanisms), *oqxA*/*oqxB* (RND efflux pumps conferring resistance to tetracyclines and fluoroquinolones), and *eptB*/*ArnT* (enzymes modifying bacterial membranes to counteract antimicrobial peptides). Notably, *Klebsiella pneumoniae OmpK37*, a porin linked to reduced beta-lactam permeability, was also widespread.

Unlike the CARD database, which includes all known ARGs, ResFinder specifically identifies acquired resistance determinants. Screening against this database (Figure 5C) did not reveal any distinct trends, as genes were distributed unevenly across the samples without a single dominant resistance determinant present in most or all cases. However, instead of isolated genes with high prevalence, this dataset featured well-represented gene groups sharing common prefixes.

The largest category included 22 *bla* genes, encoding β-lactamases that hydrolyze the β-lactam ring, conferring resistance to penicillins and cephalosporins. Tetracycline resistance was attributed to five *tet* genes—*tet(S)*, *tet(M)*, *tet(H)*, *tet(D)*, and *tet(57)*—all encoding ribosomal protection proteins that prevent antibiotic binding. A similar pattern was observed for fosfomycin resistance, with five *fosA* variants, each encoding a glutathione transferase responsible for drug inactivation.

Additional resistance mechanisms included three *qnr* genes, which protect DNA gyrase and topoisomerase IV from quinolone inhibition, and three cat genes, encoding chloramphenicol acetyltransferases that enzymatically modify chloramphenicol to prevent ribosomal binding. Aminoglycoside resistance was linked to three *aph(3′)* genes, coding for phosphotransferases that inactivate aminoglycosides through phosphorylation. Trimethoprim resistance, in turn, was associated with two *dfr* genes encoding dihydrofolate reductase variants, which bypass the drug’s inhibitory effect on folate metabolism. Notably, two *oqx* genes, previously identified in the CARD-based screening, were also detected in this dataset, reinforcing their broad distribution across samples.

Analysis of virulence factors using the VFDB database (Figure 5D) revealed notable variation across samples. NN2 and NN4 exhibited the highest number of annotated genes, while the remaining samples contained at least twice as few. Despite this overall disparity, most genes were sporadically distributed, appearing in some samples but absent in others. However, a subset of 17 genes was consistently present across all datasets, suggesting a conserved core of virulence-associated functions.

Among these, multiple genes were linked to iron acquisition, including components of the ferric enterobactin transport system (*fepA*, *fepC*, *fepD*, and *fepG*), the ferric aerobactin receptor *iutA*, and regulatory elements such as the ferric uptake regulator *fur*. Enterobactin biosynthesis and export were similarly well represented, with *entA*, *entB*, *entE*, *entF*, and *entS* forming a complete pathway for siderophore-mediated iron scavenging. Additionally, transcriptional regulation was supported by the presence of *rpoS* and *rcsB*, key players in bacterial stress response and virulence control. Other conserved factors included *ompA*, encoding an outer membrane protein involved in adhesion and immune evasion, *acrA*, a multidrug efflux pump subunit, and *fimA*, which encodes a major fimbrial subunit associated with host colonization. Notably, *galF*, encoding UTP-glucose-1-phosphate uridylyltransferase, was also uniformly detected. This enzyme plays a key role in polysaccharide biosynthesis, potentially influencing capsule formation and immune evasion strategies.

### 2.4. Genomic Characterization and Phylogenetic Placement of Identified Densoviruses

Genomic viral sequences extracted from assembled metagenomes were named *Densovirinae* sp. Isolate RBHC_NN_1–5 and *Densovirinae* sp. Isolate RBHC_VN_1–5 to reflect their origin from the Rostov Bat Rehabilitation Center (RBHC) and their respective bat hosts—*Nyctalus noctula* (NN) and *Vespertilio murinus* (VM), with the numbering indicating individual sequence variants or isolates within each group. Validation of the viral nature of these genomic sequences using VirSorter2 confirmed their 100% viral origin.

**Figure 6:**
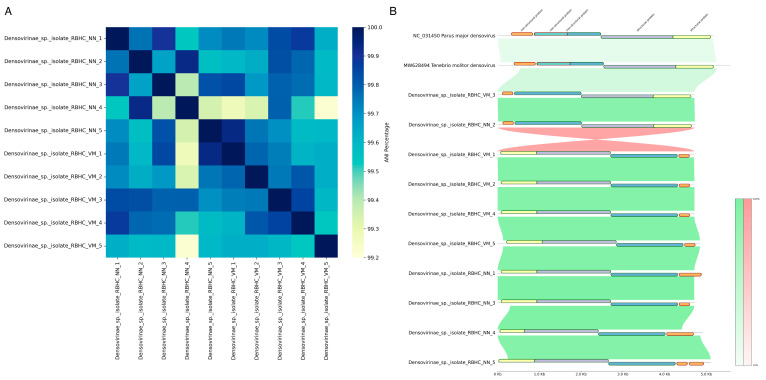
Comparative genomic analysis of densoviruses. (**A**)—Heatmap of pairwise ANI comparison of viral genomic sequences. ANI values represent the nucleotide identity between each pair of sequences, with higher values indicating greater similarity. (**B**)—Comparative genomic analysis of *Parus major densovirus*, *Tenebrio molitor densovirus*, and the *densoviruses* studied in this research. Genes included in the phylogenetic analysis are outlined in green, while those not included are outlined in red. Green connecting lines represent direct relationships between genes, while red lines indicate inverse relationships. Line color saturation reflects the degree of similarity. Gene names are displayed at the top of the graph without numbering.

Comparison of complete viral genomic sequences based on ANI (Figure 6A) revealed a high level of similarity, with a minimum identity of 99.2%. The most divergent virus was derived from sample NN4. Genome annotation using Prokka identified a genome structure consisting of two structural (SPs) and two non-structural proteins (NSPs), with one exception: the virus from sample NN5 contained two SPs and three NSPs. Gene nomenclature in our dataset may differ from that in publicly available Densovirinae genomes due to inconsistencies in NCBI RefSeq annotations. For instance, the same gene might be labeled NSP-1 in one genome and NSP-2 in another. While the numbering of genes may vary, our annotations consistently preserve their structural or non-structural classification. Comparative genomic analysis further confirmed the high degree of similarity in gene sequences across the dataset. The least similar pair, *Parus major densovirus* and *Tenebrio molitor densovirus*, still exhibited approximately 97% identity. Full-genome inversion was observed in viruses from VM1, VM2, VM4, VM5, NN1, NN3, NN4, and NN5. Additionally, the virus from NN5 displayed a duplication of the NSP-2 gene, whose length appeared to fluctuate among the studied viruses (Figure 6B). Table S1 summarizes genomic features and pairwise genomic identity of detected densoviruses.

A complete genome inversion was observed in two viral isolates (VM3 and NN1), which were mirror images of the other samples except for NN5.

**Figure 7:**
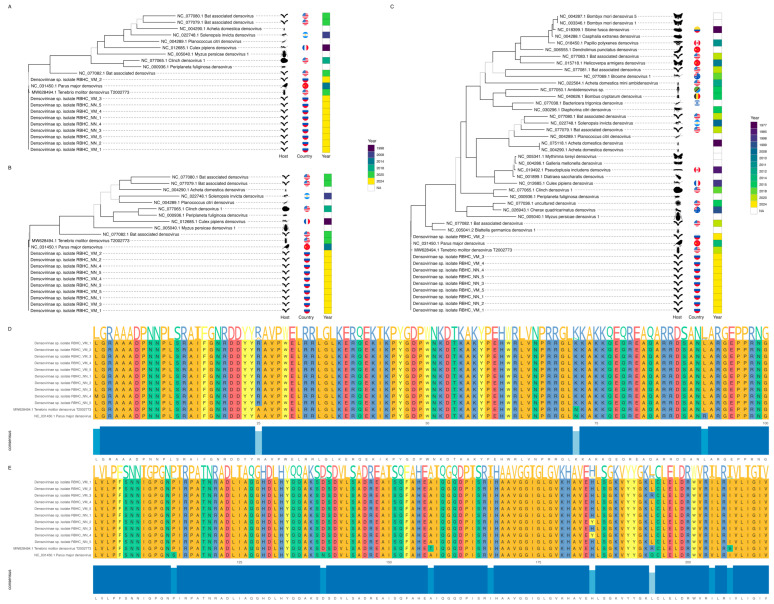
Phylogenomic analysis and protein sequence alignment. (**A**–**C**)—Maximum likelihood phylogenetic trees constructed using NSP-1 and SP-1 sequences. (**A**)—Tree based on NSP-1 and SP-1 using the Q.pfam+I+G4 substitution model. (**B**)—Tree based on SP-1 using the VT+I+G4 substitution model. (**C**)—Tree based on NSP-1 using the Q.pfam+F+I+G4 substitution model. Clades with bootstrap support below 70 are shown in gray, while those with higher support are shown in black. Trees are annotated with virus host silhouettes from the PhyloPic database, flags indicating the countries of virus isolation, and a heatmap displaying the years of isolation. (**D**,**E**)—Visualization of multiple sequence alignment of SP-2. (**D**)—Sequence region from amino acid 1 to 100. (**E**)—Sequence region from amino acid 101 to 213. Consensus sequences are shown at the top, with bar charts below each sequence representing the divergence from the consensus for each amino acid.

Orthology analysis with Proteinortho identified 45 viruses in which NSP-1 was a single-copy ortholog (10 from this study, 34 from RefSeq, and 1 from GenBank), 22 viruses with a single-copy SP-1 ortholog (10 from this study, 11 from RefSeq, and 1 from GenBank), and 12 viruses with a single-copy SP-2 ortholog (10 from this study, 1 from RefSeq, and 1 from GenBank). Separate phylogenetic trees were constructed for NSP-1 (Figure 7C) and SP-1 (Figure 7B), alongside a phylogenomic tree incorporating both genes (Figure 7A). The resulting phylogenomic tree displayed strong bootstrap support, with only three branches falling below 70%.

Across all three trees, the identified densoviruses from bat fecal metagenomes did not cluster with other bat-associated densoviruses. Instead, they consistently formed a distinct branch alongside *Parus major densovirus*. This virus was isolated from a great tit (*Parus major*) in China in 2014 [23]. Notably, *Tenebrio molitor densovirus*, which caused an outbreak with high mortality in a commercial mealworm farm [24], appeared in the same clade. Furthermore, the close relationship between *Parus major densovirus* and *Tenebrio molitor densovirus* was corroborated by an additional single-copy orthologous gene—SP-2. This gene was shared by 12 viruses, including all 10 from this study, *Parus major densovirus*, and *Tenebrio molitor densovirus*. Multiple sequence alignment of this protein (Figure 7D,E) revealed a conserved length of 213 amino acids, with only 12 amino acid variations among all 12 viruses.

## 3. Discussion

In this study, we present the first comprehensive metagenomic characterization of gut microbial communities in two synanthropic insectivorous bats, *N*. *noctula* and *V. murinus*. According to the taxonomical profiling of metagenomic data, the most remarkable signature of their gut microbiota was the dominant abundance of the reads belonging to the *Densovirus* genera. Currently, densoviruses are known to infect insects from six orders—Blattodea, Diptera, Hemiptera, Hymenoptera, Lepidoptera, and Orthoptera—as well as decapod crustaceans, such as shrimp and crayfish, and echinoderms, including sea stars [25,26]. Densoviruses cause significant damage to commercially produced invertebrate populations, including crickets, silkworms, and shrimp [27,28,29]. Many densoviruses are polytropic, exhibiting a variable host range, while others display high specificity to hosts and even organs [26]. Due to their high pathogenicity toward pests, particularly mosquitoes, densoviruses have the potential to be used as biological control agents [30]. However, the long-term impact of viral spillover on non-target native insect populations remains unclear [31]. Also, densoviruses are frequently observed in gut metagenomes of insectivorous species, particularly birds and bats. For example, Ge et al., in their metagenomic study of bat fecal samples in China, identified densoviruses among the most frequent reads and assembled contigs related to eukaryotic viruses. The authors suggested that these viruses were present in the bats’ gut virome due to the insectivorous diet of the studied bats [32]. In addition, densoviruses were detected in metagenomic sequencing of nucleic acids extracted from bat guano, feces, and gastrointestinal samples in studies in South Africa, the USA, and Croatia [33,34,35]. In all these studies, researchers also suggested the dietary origins of these viruses in the gut microbiome of bats. Given that the bats included in our study are also insectivorous, the presence of densoviruses in their gut microbiota likely originates from the consumption of infected insects. However, some findings of our bioinformatical analysis suggest that the occurrence of densoviruses in the gut microbiome of studied bats could be associated with factors beyond dietary transmission.

We assembled complete genomes of densoviruses from all studied bat-associated samples. Densovirus genomes analyzed in previous studies were composed of single-stranded linear DNA ranging from 4 to 6 kb in length and contained several major ORFs for non-structural and structural proteins [25,36]. In our research, the genome size of densoviruses was around 5 kb; nine of the densoviruses contained four ORFs, and one of them had five ORFs. According to the phylogenomic analysis targeting several genome regions of detected densoviruses, all of them appeared in one clade with *Parus major densovirus*. This virus was isolated by Yang et al. from the lung tissue of a great tit (*Parus major*) in the Jilin province of China and demonstrated to be infectious in vertebrate cell lines. The authors stated that it remains unclear whether the bird was directly infected by the densovirus or if the virus originated from insects consumed by the bird without infecting avian cells, suggesting further laboratory experiments to investigate pathogenic mechanisms in avian species [23]. Nevertheless, this virus was shown to infect vertebrate cell lines, and the high genetic homology between *Parus major densovirus* and bat-associated densoviruses from our study suggests that these viruses may also have the capacity to infect bats, potentially identifying bats as hosts for them. This hypothesis is supported by the results of the comparative genomic structure analysis, where gene inversions of identified densoviruses were observed. Some studies show that spontaneous gene inversions in the genomes of microbes increase their mutation rates, potentiating their capacity to adapt to new environments, including new hosts, and evolve virulence [37,38,39]. Although it is considered that DNA viruses have stable genomes in comparison to RNA viruses, there is an exception to this rule for DNA viruses with shorter genomes, as they often exhibit higher mutation rates due to their reduced replication fidelity and less efficient repair mechanisms [40,41]. In previous studies, densoviruses showed a high mutation rate, allowing them to adapt to new hosts, primarily invertebrates [42,43]. The majority of the studies in which the ability of densoviruses to infect vertebrate cells was investigated showed that most densoviruses exhibit limited or no active replication within vertebrate cells, pointing to the fact that their host range is limited to invertebrates [44,45]. Thus, the results of Yang et al., where *Parus major densovirus* showed the capacity to infect vertebrate cell lines, challenge the traditional notion that densoviruses are confined exclusively to invertebrate hosts [23]. Given the high genetic similarity between the *Parus major densovirus* and the densoviruses detected in bats and their different genome structures due to gene inversions, it is conceivable that these bat-associated strains may possess or be evolving the capacity to infect vertebrates, including bats themselves. Given these phylogenomic and comparative genomic analysis results, we included genomic records of densovirus isolated from commercial mealworms (*Tenebrio molitor*) from Armién et al. [24] to track the homology and genomic differences between densoviruses detected in the studied bat gut microbiota and densovirus from mealworms, which were included in the diet of bats from the Rostov Bat Rehabilitation Center. As a result, a mealworm-associated densovirus appeared in one clade with *Parus major densovirus* and the discovered bat-associated densoviruses. The mealworm-associated densovirus included in our bioinformatic analyses exhibited a high sequence identity (97–98%) with densoviruses associated with bats and birds, whereas its identity with insect-associated densoviruses was significantly lower, at approximately 50% [24], which generally corresponds to the results of our study, where this virus was phylogenetically distant from other insect-associated viruses. Thus, this genetic variant of this *Tenebrio molitor densovirus* could have evolved to increase virulence, which could have given it the ability to infect a broader range of hosts, including vertebrates such as bats and birds. However, it is important to recognize that this remains speculative and requires confirmation through comprehensive ecological monitoring and long-term surveillance of densovirus prevalence in both wild and farmed insect populations, as well as in bat populations. Such studies would help track evolutionary trends and transmission pathways. Additionally, experiments using multiple cell lines are essential to verify the ability of densoviruses to infect bats, birds, and other vertebrates, providing critical insights into their host range and pathogenic potential.

While the high homology of detected bat-associated densoviruses with *Parus major densovirus* points to the debatable ability of these viruses to infect bats due to the capability of the latter virus to infect vertebrate cells, the presence of these densoviruses in the gut microbiota of bats and their genomic variations points to the more realistic ecological impact of this finding. Bats are considered one of the most unpredictable carriers of emerging pathogens due to their ability to fly long distances and long lifespan relative to mammals of the same size, which allows them to maintain and disseminate infectious agents over broad geographic areas and extended time frames [7]. Their mobility means that pathogens can spread across different ecosystems, bypassing natural barriers that would typically limit microbe transmission. This spatial and temporal flexibility makes disease outbreaks linked to bats particularly difficult to predict and control [46]. Previously, all these features were mainly considered for emerging viruses that target humans and animals. However, the detection of densoviruses with potentially expanded host ranges in bats suggests that similar dynamics may apply to insect-associated viruses as well. If these densoviruses are capable of infecting vertebrate cells, bats could act not only as passive carriers but also as active amplifiers or reservoirs, facilitating viral adaptation and cross-species transmission. Even if bats are not the true hosts of densoviruses, their role as carriers through the gut microbiome may still contribute significantly to the environmental spread of these viruses. Through defecation, bats may introduce viable viral particles into new habitats, including agricultural fields, urban green spaces, and insect farming facilities, thereby creating opportunities for densovirus transmission to susceptible invertebrate populations. This role as ecological vectors underscores the broader influence of insectivorous bats in modulating viral circulation at the interface of natural and human-modified environments. Although our study was cross-sectional, the consistent presence and high relative abundance of densoviruses in multiple samples, combined with their genomic instability and mutation potential, suggest that the bat gut environment may support the maintenance and possibly the diversification of these viruses. Given that genomes of densoviruses detected in our metagenomic study exhibited considerable similarity to vertebrate-infecting strains and presented signs of genomic plasticity, such as gene inversions, it is reasonable to consider that the bat gut environment itself may contribute to the ongoing evolution of these viruses. Notably, this environment may act not just as a passive conduit but as an evolutionary incubator, where microbial interactions and selective pressures foster the emergence of new viral variants. This is particularly relevant for insect-targeting viruses such as densoviruses, which may undergo genetic reshuffling or host-range expansions within the bat gastrointestinal tract. Recent findings suggest that the gut microbiota of bats, particularly species such as *N. noctula*, exhibits pronounced pro-mutagenic activity, likely influencing both microbial and viral genetic variability. According to one of our previous studies, the majority of lactic acid bacteria and bacilli isolated from bat feces demonstrated increased *RecA* expression in *lux*-biosensor assays, indicative of DNA damage and activation of the SOS response system—a process known to accelerate mutation rates in both bacteria and associated viral communities [47]. This suggests that the bat gut environment, shaped by its unique microbiota, may serve as a hotspot for viral genetic diversification. As such, it could play a more active role in shaping the evolutionary trajectory of densoviruses than previously recognized.

We suggest that the dominance of densoviruses in the gut microbiome of the studied bat species is associated with their specific diet at the Rostov Bat Rehabilitation Center, which mostly consists of superworms and mealworms. This assumption is supported by the results of our phylogenomic analysis, which showed that the mealworm-infecting *Tenebrio molitor densovirus* identified by Armién et al. [24] clustered in the same clade as the densoviruses detected in the bat gut microbiome. However, it is important to note that while Armién et al. reported high mortality caused by *Tenebrio molitor densovirus* at a commercial mealworm farm, no such mortality was observed in the mealworm colony at the Rostov Bat Rehabilitation Center. This discrepancy may reflect differences in viral strain virulence, farm conditions, or host resistance. In any case, dominance in the abundance of densoviruses in the metagenomic data of the gut microbiome of the studied bat species skewed the estimates of microbial diversity. According to the alpha diversity analysis, there were no significant differences in the Shannon and Pielou indices. However, the Chao1 index indicated higher alpha diversity in the gut microbiome of *N. noctula* compared to *V. murinus*. This difference is likely due to the higher occurrence of dominant densoviruses in *V. murinus* samples in comparison to those obtained from *N. noctula* (5 vs. 1), which reduced the relative abundance of rarer taxa. Since the Chao1 index estimates species richness by giving more weight to low-abundance or rare taxa [48,49], the dominance of densoviruses in *V. murinus* likely suppressed the detection of less abundant microbes, resulting in a lower estimated richness. This conclusion is further supported by the beta diversity analysis: the PCoA plot showed that samples with densovirus dominance clustered tightly together in ordination space, suggesting that the variation between samples was largely influenced by the overwhelming abundance of a single viral species, rather than by overall differences in microbial community structure. However, it is important to note that the sample size in this study (*n* = 10) is relatively small, which limits the statistical power to detect subtle species-specific differences in microbiota composition. Therefore, conclusions regarding species-level diversity comparisons should be interpreted with caution. Nevertheless, in our previous study implementing 16S rRNA gut microbiota metaprofiling, *N. noctula* and *V. murinus* showed no significant differences in alpha diversity across Shannon, Chao1, and Pielou indices, aligning with the current shotgun results except for Chao1. Similarly, beta diversity analyses in both studies revealed overlapping microbial communities between the two species, with no significant separation by host identity once the diet was controlled for [22]. Together, these findings suggest that the gut microbiota of *N. noctula* and *V. murinus* are highly similar in both diversity and structure when not confounded by viral overrepresentation.

Our findings highlight a critical methodological consideration for future metagenomic studies of bat gut microbiota: the composition of the diet, especially in rehabilitation settings, can substantially bias microbial community profiles. In this study, bats were predominantly fed mealworms, which likely served as the source of the highly abundant densoviruses detected. This dietary skew not only resulted in an overrepresentation of densoviral reads but also masked the broader microbial diversity, as shown by alpha and beta diversity estimates. Providing a more diverse diet could minimize the dominance of single dietary-origin taxa, reduce community skew, and allow a more accurate and representative assessment of gut microbial ecosystems, reflective of wild animals. Therefore, feeding insectivorous bats with a variety of insect species under laboratory and rehabilitation center conditions is essential for obtaining robust and ecologically meaningful metagenomic profiles of their gut microbiota. While numerous studies have demonstrated that bat gut microbiome composition is strongly influenced by diet [22,50,51,52,53], the overwhelming dominance of densoviral reads observed in our metagenomic data was unexpected, even in light of these prior findings. Another option could be sampling from wild bats, as gut microbial communities of wild and captive animals differ significantly [54,55]; however, this is more challenging than conducting studies on bats in captive conditions.

Excluding densoviruses, the microbial component of the bat gut microbiota was largely dominated by members of the phylum Pseudomonadota, with notable contributions from Bacillota and Actinomycetota in several samples, which generally corresponds to previous observations [22]. At the family level, *Enterobacteriaceae* was predominant, accompanied by *Morganellaceae*, *Hafniaceae*, and to a lesser extent *Streptococcaceae* and *Drexlerviridae*. These families are commonly found in the guts of mammals and are associated with nutrient processing, host interaction, and in some cases, opportunistic pathogenicity [56,57,58,59,60,61,62]. Additionally, the presence of *Drexlerviridae*, a family of tailed bacteriophages, is noteworthy, as many of its members specifically infect *Enterobacteriaceae* [63,64,65]. These bacteriophages may play a regulatory role in shaping bacterial population dynamics within the gut, influencing microbial composition, horizontal gene transfer, and potentially contributing to the control of opportunistic pathogens [66,67,68].

Species-level analysis revealed a diverse set of enteric and environmental bacteria, including *Proteus terrae*, *Enterobacter cancerogenus*, *Klebsiella oxytoca*, *Citrobacter portucalensis*, and *Hafnia paralvei*. These taxa are consistent with prior findings in *N. noctula* from the same rehabilitation setting, where *Klebsiella*, *Citrobacter*, and *Hafnia* species were also frequently isolated, particularly from active bats prior to hibernation [69]. Their prevalence in both cultivable and metagenomic datasets supports the idea that these bacteria represent stable components of the gut community in synanthropic noctules, likely shaped by exposure to shared environmental sources such as insects, water, and human-influenced habitats. Notably, several of these taxa are recognized as opportunistic pathogens in humans and animals [70,71,72,73], suggesting a potential public health and domestic animal well-being relevance of bat-associated microbiomes. Furthermore, the detection of metabolically versatile species belonging to the *Enterobacter* and *Proteus* genera, capable of persisting in both host and non-host environments [71,74], reinforces the notion that the gut microbiota of insectivorous bats is highly adaptable and shaped by both intrinsic physiological factors and extrinsic ecological pressures.

Functional annotation of the metagenomic data revealed a relatively conserved repertoire of microbial pathways across most samples, despite the dominance of densoviruses in several of them. The most consistently detected function, peptidoglycan maturation, indicates an active community of cell wall-synthesizing bacteria, which are essential components of gut ecosystems due to their roles in colonization resistance and immune modulation [75,76,77,78]. In addition, pathways related to nucleotide biosynthesis (e.g., guanosine and adenosine deoxyribonucleotides) were recovered, though at lower prevalence, suggesting that microbial replication capacity may be constrained in low-diversity samples such as VM1 [79,80,81]. This observation is consistent with the reduced taxonomic richness and metabolic complexity in samples where densovirus dominance suppressed the representation of other taxa.

Further insights from KEGG pathway analysis revealed microbial engagement in core metabolic functions, including amino acid metabolism, carbon degradation, and energy generation via oxidative phosphorylation. Pathways associated with nitrogen and sulfur turnover, secretion systems, and biofilm formation suggest ecological versatility and adaptation to variable gut conditions [82,83,84,85]. These functions are typical of facultatively anaerobic bacteria and align with known traits of *Enterobacteriaceae* and related gut-associated taxa detected in the samples [58,86,87]. Importantly, the absence or incompleteness of functional annotations in samples like VM4 and NN3 likely reflects reduced microbial diversity due to densoviral overrepresentation, again highlighting the confounding impact of densovirus abundance.

The analysis of antibiotic resistance genes revealed a diverse resistome in the gut microbiota of both *N. noctula* and *V. murinus*, with over 500 ARGs detected across samples, and with 142 ARGs in the whole analyzed dataset. The majority of these genes encoded efflux pumps, membrane porins, and regulatory proteins associated with multidrug resistance phenotypes. The presence of *acrB*, *acrD*, *mdtB/C*, *msbA*, and *marA*, well-characterized components of the RND and major facilitator superfamily (MFS) transporters [88,89], suggests that intrinsic resistance mechanisms are prevalent within the microbial communities of these bats. These efflux systems confer resistance to a broad spectrum of antibiotics, including β-lactams, macrolides, aminoglycosides, and fluoroquinolones [90,91,92], indicating that the bat gut serves as a reservoir for multidrug resistance determinants.

Interestingly, the core resistome was conserved across samples, with *crp* identified in all metagenomes containing ARG annotations. However, samples such as NN3 and VM4, which exhibited strong densovirus dominance, showed minimal ARG representation, once again underscoring how viral overabundance can mask functional attributes of the broader microbial community. When screened against the ResFinder database, the resistome included a substantial number of *bla* genes (β-lactamases), *tet* genes (tetracycline resistance), and *fosA* variants (fosfomycin resistance), consistent with exposure to environmental or foodborne microbial communities where these ARGs are common [93,94,95,96].

The detection of virulence factors followed a similar pattern. While overall gene richness varied among samples, a core set of virulence-associated functions, particularly those related to iron acquisition (e.g., *fep*, *ent*, *iutA*), stress response (*rpoS*, *rcsB*), and adhesion (*fimA*, *ompA*), was consistently detected. These traits are hallmarks of gut-associated *Enterobacteriaceae* and suggest that members of this family not only persist in the bat gut but are potentially well-adapted to colonize and interact with host tissues [97,98,99].

In line with these observations, culture-based and molecular surveys of bat populations in different continents have repeatedly documented the same convergence of resistance and virulence traits. Multidrug-resistant *E. coli* isolated from insectivorous and frugivorous bats in southeast Nigeria, for example, carried *bla*_CTX-M-15_, *bla*_TEM_, *tet*(A), and *int1* genes together with the intimin adhesin gene *eae*, confirming that clinically important extended-spectrum beta-lactamase (ESBLs) and colonization factors can co-mobilize in bat-associated *Enterobacteriaceae* [100]. Costa et al. found that free-tailed bats (*Tadarida brasiliensis*) from Southern Brazil harbor enterococci carrying *ermC*, *tetM,* and *vanC1/2/3* together with virulence genes *gelE* and *ace* (with occasional *agg*, *cylA*, *esp*), highlighting the tight linkage of resistance and pathogenic traits in bat-associated Gram-positive commensals [101]. European free-tailed bats (*Tadarida teniotis*) in Portugal hosted ESBL-*E. coli* in about 10% of fecal samples; isolates carried *bla*_CTX-M-1/-3_, often with *bla*_SHV_, *bla*_TEM,_ or *bla*_OXA_, as well as *tet*(A)/*tet*(B), and invariably the *fimA* adhesin, again coupling mobilizable β-lactamases with colonization factors in bat-borne *Enterobacteriaceae* [102]. Lesser horseshoe bats (*Rhinolophus monoceros*) from India, likewise, yielded gut isolates resistant to β-lactams such as penicillin G and cefoxitin, suggesting underlying β-lactamase ARGs, and *Bacillus* and *Pseudomonas* species from the same samples produced hemolysin and protease, coupling antimicrobial resistance with clear virulence functions [103]. Similarly, a metagenomic survey by Huang et al. revealed over a thousand ARGs across wild bats with diverse diets in China, with multidrug and polymyxin resistance genes being particularly prevalent. High-risk ARGs were genetically linked to mobile elements on zoonotic pathogens, and their abundance and diversity were diet-dependent, most pronounced in carnivorous and sanguivorous bats, suggesting that feeding ecology shapes both the resistome and associated risks. This study further emphasizes bats as potent reservoirs and vectors of clinically relevant resistance traits with mobilization potential [104].

Collectively, these findings support the growing recognition of synanthropic bats as environmental reservoirs of both antimicrobial resistance and virulence traits. While direct transmission risks to humans or animals remain speculative, the co-occurrence of ARGs and virulence genes within mobile or pathogenic bacterial taxa poses a potential One Health concern, especially in urbanized or agricultural interfaces where bats and humans frequently interact. Additionally, the detection of clinically relevant resistance genes in wildlife microbiomes further supports calls for expanded surveillance of resistomes beyond traditional agricultural and hospital environments.

## 4. Materials and Methods

### 4.1. Sampling

Sampling was conducted in October 2022 at the Rostov Bat Rehabilitation Center (Don State Technical University, Rostov-on-Don, Russia) for metagenomic investigation of gut microbiota signatures. For these studies, fecal samples were collected from common noctules (*Nyctalus noctula* [*n* = 5, NN1–5]) and parti-colored bats (*Vespertilio murinus* [*n* = 5, VM1–5]). Bats included in this research were in the active state, seized from households of Rostov-on-Don at the owners’ request, and then arrived at the bat rehabilitation facility, where sampling was performed after their arrival and feeding with superworms and mealworms. Handling of animals at the bat rehabilitation center was approved by the Don State Technical University local ethics committee (Protocol No. 5, 2022). A minimum of 0.5 g of fecal samples were collected from bats into sterile 1.5 mL tubes, snap-frozen, and then stored at −80 °C.

### 4.2. DNA Extraction and High-Throughput Sequencing

Extraction of DNA was performed with a DNA-Sorb B kit (AmpliSens, Moscow, Russia) according to the manufacturer’s protocol. Libraries were prepared using the NEBNext Ultra II DNA Library Prep Kit (New England Biolabs, Beverly, MA, USA) and sequenced using the Illumina NovaSeq 6000 sequencing system according to the manufacturer’s instructions (Illumina, San Diego, CA, USA) with the generation of 2 × 150 bp reads.

### 4.3. Quality Control of Raw Reads and Taxonomic Classification

The quality of raw sequencing reads was assessed using FastQC v.0.12.1 (https://www.bioinformatics.babraham.ac.uk/projects/fastqc/, accessed on 10 May 2025), followed by report aggregation for each paired-end sample using MultiQC v.1.23 [105]. Taxonomic classification of microbial communities in metagenomic DNA samples was performed using Kraken2 v.2.1.3 [106] with the PlusPF database (Standard plus RefSeq protozoa & fungi; 28 December 2024). Taxon abundance at the genus and species levels was re-estimated using Bracken v.3.1 to improve classification accuracy [107]. The resulting taxonomic profiles were formatted into structured tables using KrakenParser v.0.1.51 (https://github.com/PopovIILab/KrakenParser, accessed on 10 May 2025).

### 4.4. Diversity and Differential Abundance Analysis

Taxonomic composition profiles were visualized as bar charts representing the relative abundance of microorganisms across samples at the phylum, family, and species levels. To assess biological diversity, both alpha and beta diversity metrics were calculated. The visualizations were generated in R using the tidyverse, ggtext, patchwork, paletteer, data.table, tibble, dplyr, tidyr, ggplot2, and scales packages.

Alpha diversity was quantified using the Shannon [108], Pielou [109], and Chao1 [110] indices. Differences in alpha diversity between the two bat species were evaluated using the Mann–Whitney U test [111], with results visualized as box plots. Beta diversity was assessed using the Bray–Curtis [112] and Jaccard [113] indices, with statistical comparisons performed using permutational multivariate analysis of variance (PERMANOVA) [114]. Principal coordinates analysis (PCoA) plots were used to visualize beta diversity patterns. Alpha and beta diversity metrics were visualized in R with tidyverse, ggtext, patchwork, vegan, glue, and scales.

To identify taxa with significantly different abundance levels between *N. noctula* and *V. murinus*, differential abundance analysis was conducted using MaAsLin2 [115]. The analysis employed a linear model with total sum scaling (TSS) normalization and Benjamini–Hochberg correction for False Discovery Rate control [116], using the following parameters: *min_prevalence = 0.01*, *min_abundance = 50*,*000*, *analysis_method = LM*, *max_significance = 0.05*. Results were visualized as volcano plots generated in R using the tidyverse, ggtext, and ggrepel packages.

### 4.5. Metagenome Assembly and Functional Profiling

Metagenome assembly was performed using metaSPAdes v.4.0.0 [117], and assembly quality was assessed with metaQUAST v.5.2.0 [118]. To minimize noise and improve downstream analyses, contigs shorter than 3500 nucleotides were filtered out after quality evaluation.

Functional annotation of metabolic pathways was conducted using HUMAnN3 v.3.9 [119], a tool designed for precise quantitative assessment of functional potential. Annotation relied on the ChocoPhlAn and UniRef databases [120]. HUMAnN3 results were merged and normalized via total sum scaling, yielding relative pathway abundances across metagenomes. All unmapped, unintegrated, and unclassified values were removed to ensure clarity. Pathway abundances were visualized as heatmaps using tidyverse, ggplot2, ggtext, patchwork, and vegan. Differential pathway abundance was further assessed with MaAsLin2 v.1.7.3 [115] using identical model parameters but omitting normalization, as it had already been applied. Volcano plots were generated to illustrate significant differences in pathway abundance between groups.

In addition, microbial community functional annotation was performed using eggNOG-mapper v.2.1.12 [121]. The analysis was optimized for metagenomic inputs, employing Prodigal v.2.6.3 [122] for gene recognition. To assess the completeness of KEGG orthologous group (KO)-based metabolic pathways, annotation results were processed with KEGGaNOG v.0.7.42 (https://github.com/iliapopov17/KEGGaNOG, accessed on 10 May 2025), a custom utility designed to decode KEGG_ko values via KEGG Decoder [123]. KEGGaNOG generated heatmaps representing pathway completeness across metagenomes. To compare KEGG pathway completeness between samples, a correlation matrix was constructed, retaining only correlations above 0.7 for visualization clarity. The correlation network was rendered to highlight key differences between samples.

The dual approach to functional annotation—HUMAnN3 for pathway-level integration and quantification, and eggNOG-mapper for broader gene annotation—ensured a comprehensive functional characterization of the metagenomes.

Finally, assembled metagenomes were screened for antibiotic resistance genes (ARGs) and virulence factors using ABRicate v.1.0.1 (https://github.com/tseemann/abricate, accessed on 10 May 2025), leveraging the CARD [124], ResFinder [125], and VFDB [126] databases. The distribution of identified genes across samples was visualized using bar plots, while presence/absence patterns were represented as heatmaps. The identification of the numbers of overlapping and non-overlapping genes by different databases was visualized using ggVennDiagram [127]. All visualizations were implemented in R using tidyverse, ggtext, patchwork, paletteer, data.table, tibble, dplyr, tidyr, ggplot2, and scales.

### 4.6. Genomic Characterization and Phylogenetic Analysis of Densoviruses

Given the high relative abundance of *Parus major densovirus* in most analyzed samples, as identified by Kraken2, densoviruses were subjected to an in-depth investigation.

Complete genomes of all known densoviruses were retrieved from the NCBI RefSeq database [128] using Entrez-direct [129], with the following query: Densovirus AND “complete genome” AND srcdb_refseq[PROP]. A custom BLAST v.2.16.0 database [130] was then constructed using 44 densovirus genomes from RefSeq. All metagenome assemblies were screened against this local database to extract viral sequences. To validate BLAST hits and confirm viral origin, VirSorter2 v.2.2.4 [131] was employed, ultimately identifying 10 near-complete densovirus genomes from the metagenomic data.

To assess their genomic similarity, pairwise Average Nucleotide Identity (ANI) was calculated using FastANI v.1.34 [132], and the results were visualized as a heatmap using pandas, matplotlib, and seaborn Python libraries. Genome annotation was performed with Prokka v.1.14.6 [133], incorporating protein sequences of densoviruses from the NCBI Protein database (https://www.ncbi.nlm.nih.gov/protein/, accessed on 10 May 2025). The annotation results served as the foundation for further phylogenomic analysis.

The phylogenomic framework included proteomes of the 10 newly identified densoviruses, the 44 RefSeq genomes, and an additional densovirus described by Armién et al. [24]. Orthologous gene identification was conducted using Proteinortho v.6.3.2 [134], retaining only single-copy orthologs for downstream analysis. Two orthologous genes were selected for phylogenetic inference, while an additional gene, detected in a limited number of species, was used solely for sequence alignment visualization.

Multiple sequence alignment was performed using MAFFT v.7.526 [135], with substitution models determined via ModelFinder [136]. Maximum likelihood phylogenies were reconstructed with IQ-TREE2 v.2.3.6 [137], incorporating 1000 ultrafast bootstrap replicates [138]. Separate trees were generated for the two genes, along with a combined dataset tree integrating both. Phylogenetic trees were visualized and annotated using ggtree [139], incorporating metadata on host species, geographic origin, and year of isolation. Host taxa were represented by silhouettes sourced from PhyloPic (https://www.phylopic.org, accessed on 10 May 2025). Annotation data were retrieved from NCBI metadata using Phyloki v.0.5.51 (https://github.com/iliapopov17/phyloki, accessed on 10 May 2025). Sequence alignment was visualized with ggmsa [140].

Finally, genome architectures of the densoviruses were compared using pyGenomeViz v.1.5.0 (https://github.com/moshi4/pyGenomeViz, accessed on 10 May 2025). Prokka annotation results were integrated with Proteinortho data to generate a consensus gene map. Viral genomes were aligned vertically, displaying all identified genes. Genomic synteny was assessed using MUMmer v.3.23 [141], with collinear regions depicted as linking bands to highlight homologous segments across genomes.

### 4.7. Data Analysis and Visualization

Throughout the data analysis process, Snakemake pipelines [142] were extensively employed to automate and parallelize tasks. Data transformation and visualization steps were carried out using custom scripts written in Python (version 3.12), R (version 4.4.2), and bash.

## 5. Conclusions

This study provides novel metagenomic insights into the gut microbiota of two synanthropic insectivorous bat species, *Nyctalus noctula* and *Vespertilio murinus*, emphasizing their ecological roles as reservoirs and potential disseminators of diverse microbial and viral taxa. While the bacterial communities were dominated by facultative anaerobes such as Enterobacteriaceae, which are important for public health and domestic animal welfare, the most striking finding was the consistently high abundance of densoviruses, particularly those closely related to Parus major densovirus, known for its infectivity in vertebrate cell lines.

This observation raises the possibility that bats, long recognized as carriers of zoonotic viruses affecting mammals, may also serve as previously overlooked hosts for insect-targeting viruses, including highly pathogenic strains. The detection of gene inversions and high genomic similarity to densoviruses implicated in lethal outbreaks in farmed insects suggests that the bat gut environment may not only preserve but also promote the evolution and potential host-range expansion of these viruses. This is further supported by the previously investigated pro-mutagenic potential of bat gut microbiota, which may act as a hotspot for viral diversification and adaptation.

In addition to their viral ecology, bats in this study harbored a broad and diverse repertoire of antimicrobial resistance genes, including multidrug efflux pumps, β-lactamases, and tetracycline and fosfomycin resistance determinants. These findings align with the growing recognition of wildlife as a reservoir of clinically relevant ARGs, with synanthropic bats potentially contributing to the environmental spread of resistance through fecal shedding in urban and agricultural ecosystems. The co-occurrence of ARGs and virulence factors within gut-associated bacterial communities further underscores the One Health implications of bat microbiomes.

Taken together, our findings call for a broader ecological reassessment of bats, not only as key players in the emergence of mammalian pathogens but also as active participants in both the circulation and emergence of insect-targeting viruses and antimicrobial resistance genes. Future research should integrate longitudinal sampling, functional validation, and virome-focused approaches to elucidate the full spectrum of microbial and viral dynamics within bat populations, particularly in the context of rapidly changing anthropogenic environments.

## Data Availability

The raw sequencing data used for this study are available in the National Center for Biotechnological Information’s Short Read Archive under BioProject PRJNA1253095. The pipeline used for the in silico and bioinformatic data analysis has been deposited in GitHub: https://github.com/PopovIILab/BatShotMetaFlow (accessed on 10 May 2025).

## Supplementary Materials

The following supporting information can be downloaded at https://www.mdpi.com/article/10.3390/ijms26135941/s1.

## Abbreviations

The following abbreviations are used in this manuscript:

ARGs	Antibiotic Resistance Genes
VFDB	Virulence Factors of Bacterial Database
CARD	Comprehensive Antibiotic Resistance Database
PCoA	Principal Coordinates Analysis
ANI	Average Nucleotide Identity
ORFs	Open Reading Frames
NSP	Non-Structural Protein
SP	Structural Protein
RND	Resistance-Nodulation-Cell Division
MFS	Major Facilitator Superfamily
KEGG	Kyoto Encyclopedia of Genes and Genomes
KO	KEGG Orthologous
PERMANOVA	Permutational Multivariate Analysis of Variance
DNA	Deoxyribonucleic Acid
RNA	Ribonucleic Acid
