# Supplementary material for: Metagenomic Investigation of Intestinal Microbiota of Insectivorous Synanthropic Bats: Densoviruses, Antibiotic Resistance Genes, and Functional Profiling of Gut Microbial Communities
Source: Int J Mol Sci. 2025 Jun 20;26(13):5941. doi: 10.3390/ijms26135941 (PMC12250462; doi:10.3390/ijms26135941)
Supplement: Supplementary file 1 [file ijms-26-05941-s001.zip › Supplementary.pdf]

Supplementary figures

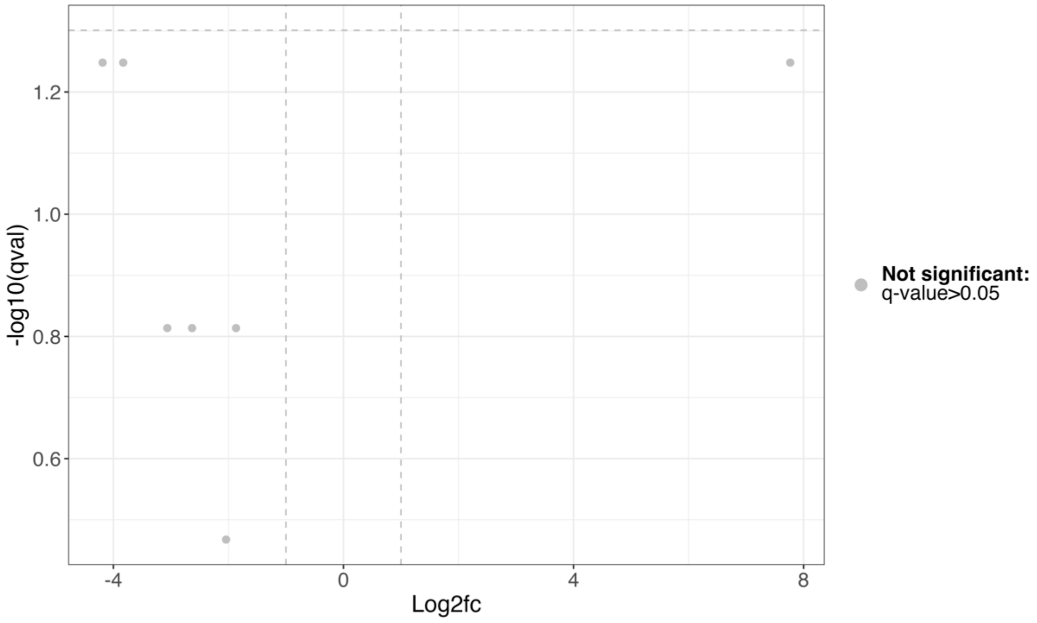

Figure S1. Results of the differential abundance analysis of gut microbial communities of *Vespertilio murinus* and *Nyctalus noctula* on the phylum level.

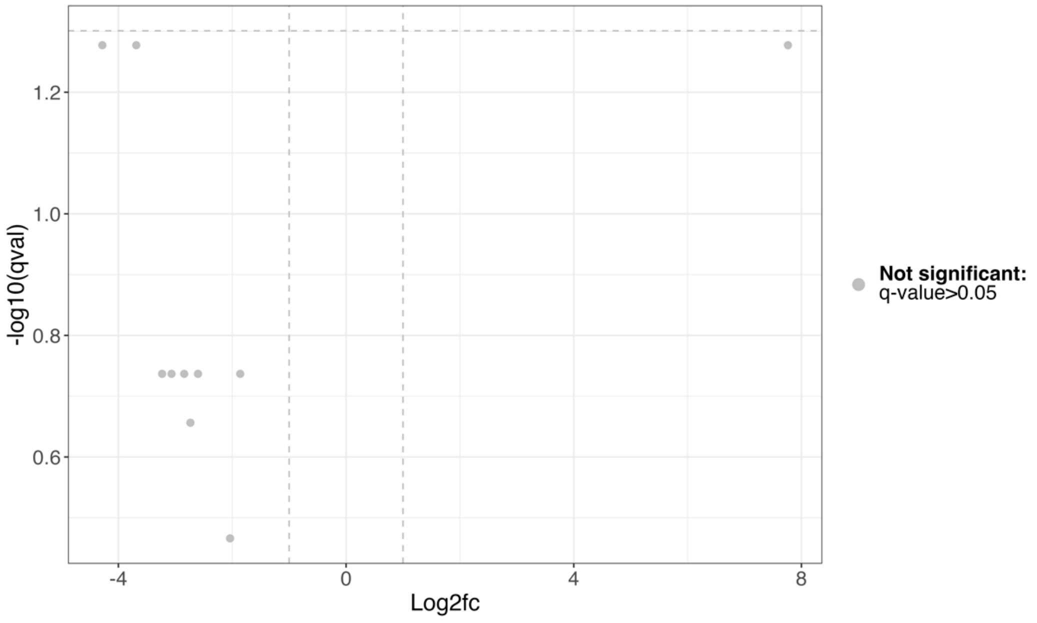

Figure S2. Results of the differential abundance analysis of gut microbial communities of *Vespertilio murinus* and *Nyctalus noctula* on the class level.

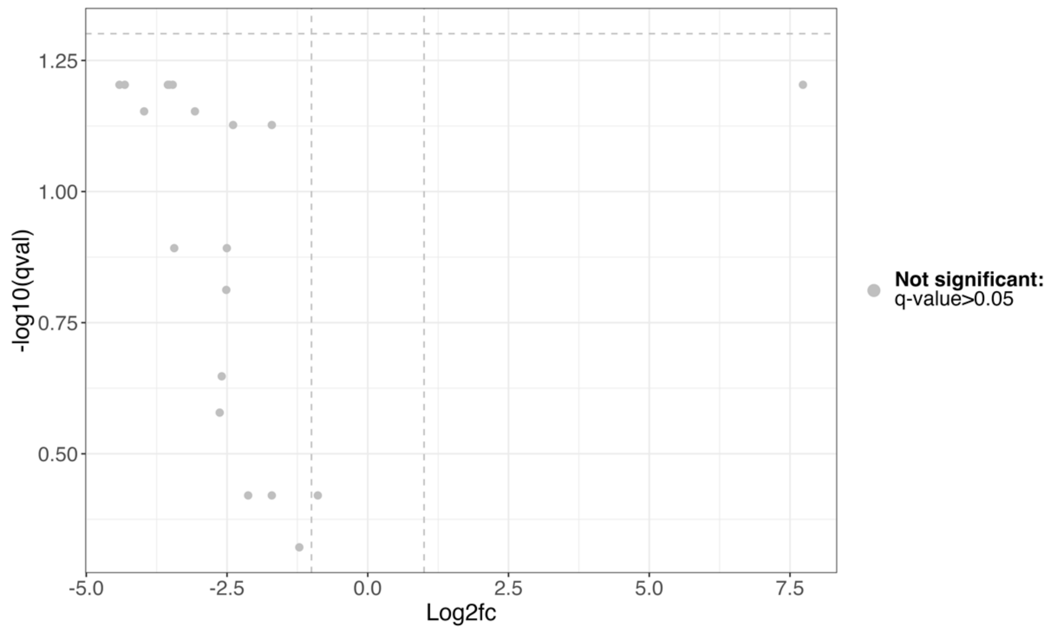

Figure S3. Results of the differential abundance analysis of gut microbial communities of *Vespertilio murinus* and *Nyctalus noctula* on the order level.

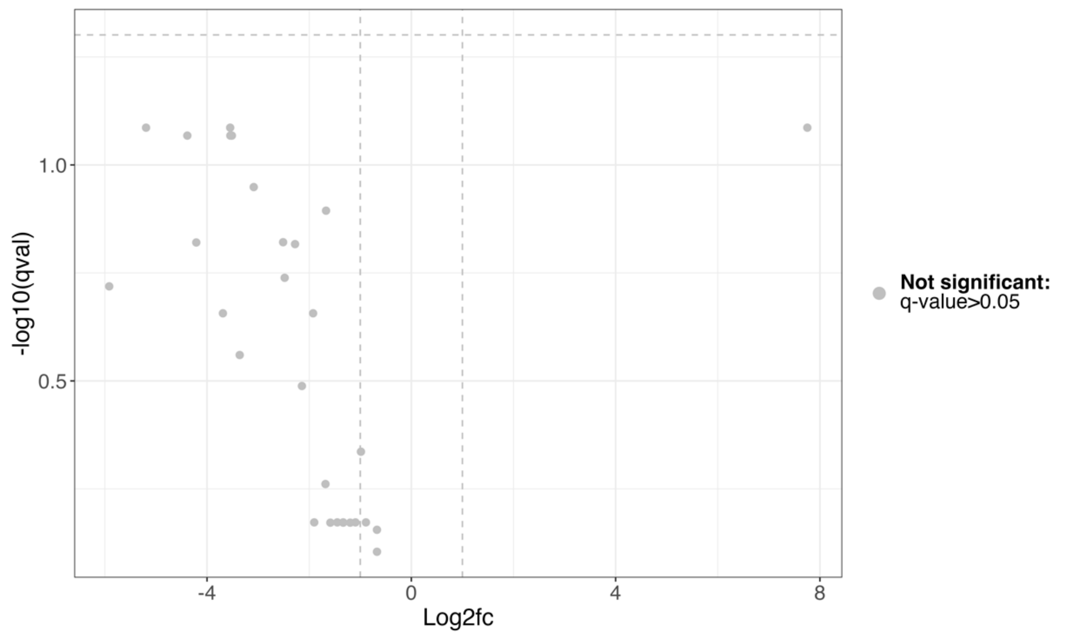

Figure S4. Results of the differential abundance analysis of gut microbial communities of *Vespertilio murinus* and *Nyctalus noctula* on the family level.

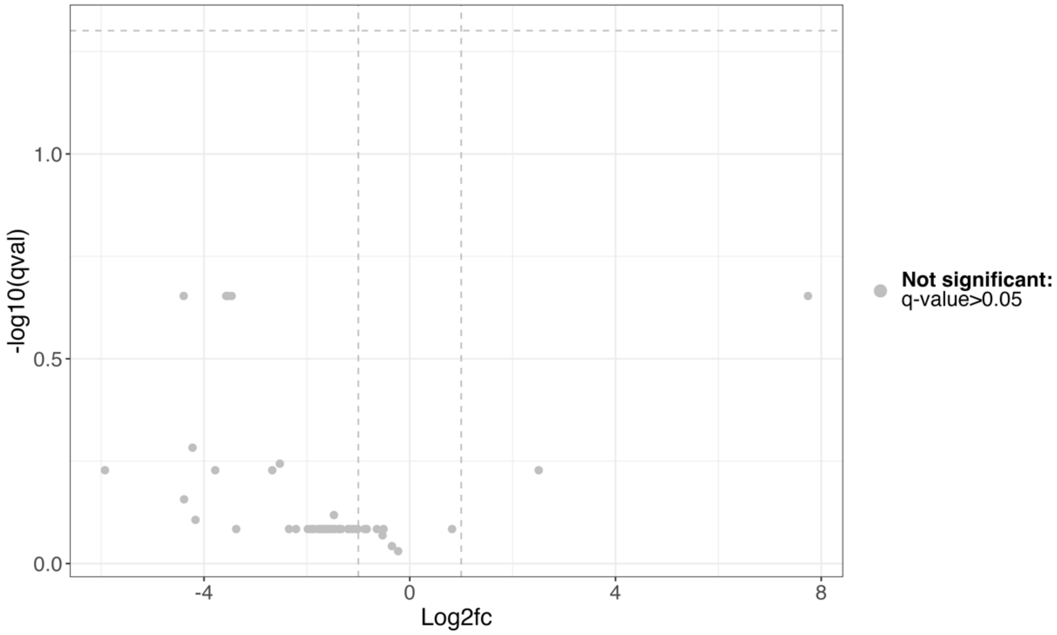

Figure S5. Results of the differential abundance analysis of gut microbial communities of *Vespertilio murinus* and *Nyctalus noctula* on the genus level.

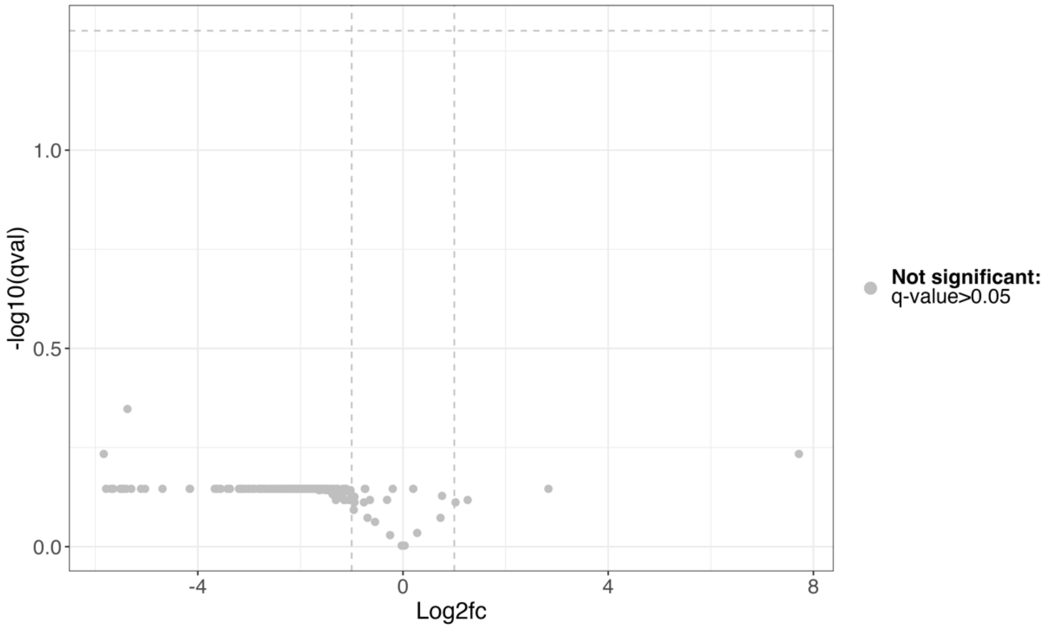

Figure S6. Results of the differential abundance analysis of gut microbial communities of *Vespertilio murinus* and *Nyctalus noctula* on the species level.

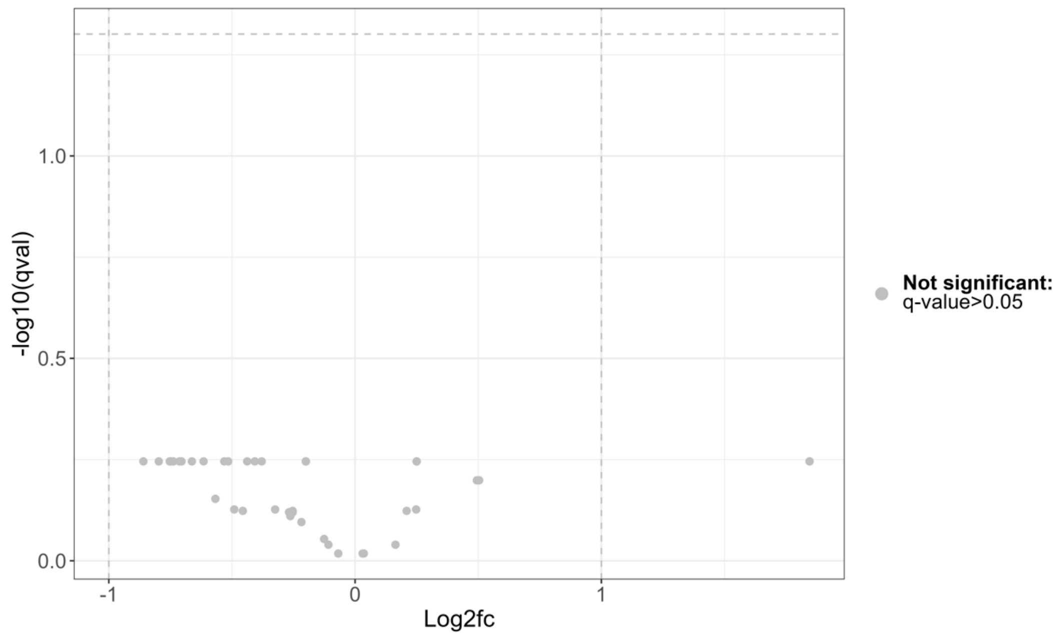

Figure S7. Results of the differential abundance analysis of metabolic pathways of gut microbiota of *Vespertilio murinus* and *Nyctalus noctula*.
